# Hypercapnia: An Aggravating Factor in Asthma

**DOI:** 10.3390/jcm9103207

**Published:** 2020-10-05

**Authors:** Masahiko Shigemura, Tetsuya Homma, Jacob I Sznajder

**Affiliations:** 1 Division of Pulmonary and Critical Care Medicine, Northwestern University, Chicago, IL 60611, USA; masahiko.shigemura@northwestern.edu; 2 Department of Allergology and Respiratory Medicine, University of Showa School of Medicine, Tokyo 142-0064, Japan; oldham726@yahoo.co.jp

**Keywords:** asthma, respiratory failure, hypercapnia, airway contractility, innate immune response, obesity

## Abstract

Asthma is a common chronic respiratory disorder with relatively good outcomes in the majority of patients with appropriate maintenance therapy. However, in a small minority, patients can experience severe asthma with respiratory failure and hypercapnia, necessitating intensive care unit admission. Hypercapnia occurs due to alveolar hypoventilation and insufficient removal of carbon dioxide (CO_2_) from the blood. Although mild hypercapnia is generally well tolerated in patients with asthma, there is accumulating evidence that elevated levels of CO_2_ can act as a gaso-signaling molecule, triggering deleterious effects in various organs such as the lung, skeletal muscles and the innate immune system. Here, we review recent advances on pathophysiological response to hypercapnia and discuss potential detrimental effects of hypercapnia in patients with asthma.

## 1. Introduction

Asthma is a chronic disease characterized by reversible airway obstruction caused by bronchial smooth muscle contraction, airway inflammation and increased secretions, and is currently estimated that more than 330 million people are afflicted with this disease worldwide [1,2]. The natural history of asthma is punctuated by acute exacerbations, most of which respond to conventional treatment using inhaled bronchodilators and corticosteroids, and oxygen. However, deterioration or failure to respond to these measures sometimes leads to severe respiratory failure. Approximately 5 to 10% of asthmatic patients experience a severe asthma attack each year and, of those who are admitted to hospital, 10% require intensive care unit (ICU) admission [3]. Ten to twenty-six percent of cases with acute severe asthma present to the emergency department with hypercapnia [3]. In-hospital mortality rates for patients with severe asthma who require ICU admission is 3.2 to 9.8%, with higher mortality rates seen in those patients who require invasive ventilation [4]. Hypercapnia is associated with the institution of mechanical ventilation and greater risk for in-hospital death in acute severe asthma [5,6].

Hypercapnia, an elevation in the arterial carbon dioxide (CO_2_) tension, is a complication of inadequate alveolar gas exchange in patients with severe acute and chronic lung diseases [7] including asthma [5,6,8,9,10,11,12,13,14,15,16,17]. It has been initially reported that hypercapnia was innocuous or even protective in mechanically ventilated patients with severe asthma [9,10], acute lung injury and acute respiratory distress syndrome (ARDS) [18,19,20,21], where the concepts of “permissive” and even “therapeutic” hypercapnia have been proposed for the mechanically ventilated patients. The lower tidal volumes during protective ventilation can lead to hypercapnia and an associated drop in pH resulting in hypercapnic respiratory acidosis that has been reported as a protective effect via the inhibition of the nuclear factor-κB (NF-κB) pathway, a pivotal transcription activator in inflammation and injury [7,22]. However, in recent years, it has become increasingly evident that elevated CO_2_ acts as a gaso-signaling molecule, resulting in deleterious effects in various organs such as the lung [23,24,25,26,27,28,29] and skeletal muscles [30,31,32] as well as innate immunity system [25,29,33,34,35,36,37]. In the lung, recent studies reported that high levels of CO_2_ activate specific gene expression [26,38,39,40] and signal transduction pathways with adverse consequences on alveolar fluid clearance through Na, K-ATPase endocytosis via intracellular calcium- or extracellular signal-regulated kinase (ERK)-mediated AMP-activated protein kinase (AMPK)/protein kinase C-ζ/c-Jun-N-Terminal Kinase (JNK) signaling or soluble adenylyl cyclase-mediated protein kinase A-Iα signaling [24,41,42,43,44,45,46] and epithelial cell repair via AMPK-mediated Rac1-GTPase signaling, NF-κB pathways or miR-183-regulated mitochondrial dysfunction [28,47,48,49]. In addition, a secondary analysis of three prospective non-interventional cohort studies of ARDS patients receiving mechanically ventilation reported that severe hypercapnia was independently associated with higher ICU mortality [23]. These recent reports have led to the re-evaluation of the potential complexity of hypercapnia effects, and are stimulating more research to better understand its biologic effects. Here, we review recent advances on pathophysiological response to hypercapnia; CO_2_ sensing, CO_2_-dependent regulation of respiration and signaling events initiated by hypercapnia, and discuss the relevance of these data to patients with asthma and hypercapnia.

## 2. CO_2_ Sensing and Respiration

Cells possess the ability to sense and respond to changes in concentration of gaseous molecules through evolutionarily conserved pathways [50]. CO_2_ is a small non-polar molecule and produced in the mitochondria of eukaryotic cells during oxidative phosphorylation and its physiological levels in mammalian tissues (~5%) [7] are significantly higher than those found in the atmosphere (~0.04%) [51,52]. CO_2_ is thought to traverse biological cell membranes via passive diffusion, depending upon the transmembrane concentration gradient of CO_2_ and the lipid/water partition behavior of the gas [53]. However, the discovery of the effect of cholesterol on CO_2_ permeability and of protein channels used by CO_2_ to cross membranes such as aquaporins (AQPs) [54,55] and rhesus proteins [56] has challenged this view. Functionally, AQP1, AQP4-M23, AQP5 and AQP6 seem to effect high permeability for CO_2_ [55]. In the lung, AQP1 is expressed in microvascular endothelia, AQP3 and AQP4 in airway epithelia, and AQP5 in type I alveolar epithelial cells and a subset of airway epithelial cells [54]. Once inside the cell, CO_2_ very rapidly equilibrates with its hydrated form, H_2_CO_3_, which in turn dissociates into H^+^ and HCO_3_^−^ catalyzed by carbonic anhydrases [57].

CO_2_ + H_2_O ⇌ H_2_CO_3_ ⇌ H^+^ + HCO_3_^−^

Increased partial pressure CO_2_ (pCO_2_) in the blood, which occurs due to insufficient removal of CO_2_ (alveolar hypoventilation), can give rise to elevated pCO_2_ in the cerebrospinal fluid and result in elevated H^+^ concentrations, cerebrospinal fluid acidification. Multiple sites within the central nervous system are capable of sensing and eliciting rapid adaptive responses to these changes, which results in alteration in the rate and depth of breathing.

### 2.1. Central CO_2_ Chemosensing

Acute chemosensing of CO_2_ is a complex process involving integration of multiple brain regions, effector channels and molecules. Neurons detect changes in CO_2_/H^+^ and modulate the CO_2_-chemosensory regulation of respiration. Several regions in the brain, particularly medulla oblongata, medullary raphe and cerebellum in the brain stem, have been described as potential area of interest [58,59,60,61,62]. A recent report suggests important crosstalk between the carotid body and central chemosensing regions of the brain that determines the respiratory response to altered CO_2_ level [63]. Much of the work in this area has been reviewed by Cummins et al. [50]. Here, we review key molecular mechanisms of the brain that are involved in CO_2_ chemosensitivity to elicit the change in respiratory rate.

#### 2.1.1. pH-Sensitive Ion Channel

The ability to acutely sense and respond to elevated CO_2_ levels occurs via a physiological adaptation to reflect acid/base balance in the blood. The pH is a major effector of CO_2_-dependent signaling in the brain. The Twik-related acid-sensing potassium (K^+^) (TASK) channels are members of the background K_2P_ channel family that facilitate selective K^+^ leak and contribute to the negative resting membrane potential in cells [64]. TASK-1, -2 and -3 channels play a role in CO_2_-dependent regulation of breathing. TASK-1 and -3 channels display acid sensitivity and are widely expressed in known chemosensing regions in the brain [65]. Catecholaminergic neurons in the locus coeuruleus have also been proposed to contribute to the ventilatory response to hypercapnia [66]. Specifically, several of transient receptor potential channels (TRPC), in particular TRPC 5, are sensitive to pH and enriched in chemosensory regions of the brain.

#### 2.1.2. CO_2_-Sensitive Connexin Protein

Huckstepp et al., reported adenosine triphosphate (ATP) released from brain slices derived from the ventral surface of the medulla oblongata in response to elevated CO_2_ levels, independently of extracellular acidification [67]. Connexin hemi-channels including connexin 26 in the medulla oblongata were also reported as contributing to the ATP release in known chemosensory regions. Subsequent studies revealed a role for inward rectifying K^+^ channels [67], participating in hyperpolarization of excitable cells and CO_2_-dependent inhibition during hypercapnia. A recent study reported that the chemo-sensitivity of connexin 26 linked directly to a CO_2_-dependent posttranslational modification of the channel, independently of pH changes [68]. Molecular CO_2_ can bind to Lys125 on connexin 26 forming a carbamate bridge between Lys125 and a neighboring residue, Arg104. The CO_2_-dependent modification causes a structural change in the gap junction, which facilitates altered connexin-dependent signaling (e.g., ATP release). This study identified central chemo-sensitivity to elevated CO_2_ mediated not only by indirect changes in pH but directly by high CO_2_-dependent modifications.

## 3. Hypercapnia in Asthmatic Patients

The pH-modulating effects of hypercapnia can be attenuated via bicarbonate reabsorption by the kidneys [69]. However, during acute hypercapnia, the buffering capacity of the blood is not sufficient to handle the excess CO_2_, resulting in acute respiratory acidosis (pH < 7.35). Levels of partial pressure of CO_2_ in arterial blood (PaCO_2_) among asthmatic patients experiencing severe exacerbations varies considerably. Scala reported that hypercapnia occurred in 10 to 26% of cases presenting to the emergency department with greater airway obstruction, higher respiratory rate and pulsus paradoxus [3]. The medical literature reports cases of severe hypercapnia in asthmatic patients with values of PaCO_2_ reaching 202 and 218 mmHg (pH value, 6.68 and 6.90, respectively) in two 24 and 28 year-old women during severe status asthmaticus [12], 208 mmHg (pH = 6.73) in a 35 year-old woman during an episode of near fatal asthma [13] and 175 mmHg (pH = 6.99) in a 33 year-old woman during severe status asthma [17]. Pediatric reports are scarce, but an eight year-old boy during an episode of near fatal asthma had PaCO_2_ values of 293 mmHg (pH = 6.77) [14], a two year-old girl with status asthmaticus had the values of 238 mmHg (pH = 6.71) [16] and an eleven year-old boy with status asthmaticus with 187 mmHg and pH of 6.84 [15].

In patients with asthma, the presence of hypercapnia reflects more severe airflow obstruction and more severe chronic asthma conditions [8,11]. Mountain et al., reported that acute asthmatic patients with hypercapnia (mean PaCO_2_ value, 53.6 mmHg) were more likely to require maintenance therapy with β-adrenergic agents and corticosteroids, and were less likely to have been discharged from an emergency room visit [11]. In a secondary analysis of data from a clinical database, the Intensive Care National Audit and Research Centre (ICNARC), hypercapnia was shown to be associated with severe asthmatic patients requiring mechanical ventilation and greater risk for in-hospital death after adjusting for Acute Physiology and Chronic Health Evaluation (APACHE) II score [5]. Stow et al., reported that in asthmatic patients admitted to Australian ICUs from 1996 to 2003 non-survivors who were not ventilated in the first 24 h had a higher PaCO_2_ level, but no difference of arterial oxygen tension, than those who did survive (mean PaCO_2_ value, 74.1 vs. 54.3 mmHg) [6]. Asthmatic patients presenting with worsening hypercapnia and respiratory acidosis require intubation and the need for ventilatory assistance. In mechanically ventilated patients with asthma, “permissive” hypercapnia is the currently recommended strategy for severe asthma with the goals of minimizing barotrauma/volutrauma [9,10,70,71]. Elsayegh et al., reported that the peak value of PaCO_2_ on the first day of mechanical ventilation with the “permissive” hypercapnia averaged 67 mm Hg and exceeded 100 mmHg in 12% of cases (the highest PaCO_2_ value, 159 mmHg) [72]. With this approach, decreasing the respiratory rate and tidal volume as well as increasing the inspiratory flow rate leads to an increase in expiratory time and decrease of the dynamic hyperinflation.

## 4. Detrimental Effects of Hypercapnia in Asthma

The symptoms and physiologic consequences of hypercapnia are significant. A series of adaptive mechanisms are activated in vital organs such as brain and heart to preserve tissue oxygenation and perfusion, in particular by preservation and defense of intracellular pH. The injurious effects of hypercapnia on the central nervous and cardiovascular systems are well documented [70]. Hypercapnia results in cerebrovascular vasodilatation leading to an increase in intracranial pressure by increasing the blood volume in the brain [70,73]. There have been reports in patients with severe asthma developing cerebral edema and subarachnoid hemorrhage as a complication of hypercapnia or “permissive hypercapnia” [15,17,74]. The myocardial response to hypercapnia is characterized by impairment in contractility due to acute respiratory acidosis, which is reversible [70,75]. Accumulating scientific evidence points to the role of high CO_2_ on the lung airways, innate immunity and adipogenesis, which could contribute to the disease pathogenesis and progression of asthma.

### 4.1. Lung Airways

The predominant feature of asthma is shortness of breath or dyspnea due to the excessive constriction of the airway smooth muscles. As such, relieving airway smooth muscle constriction is a therapeutic target of asthma management. Elevated CO_2_ levels are reported to modulate the tone of lung airways, which is in a dynamic equilibrium between excitatory and inhibitory mechanisms. Lung airway cells sense and respond to changes in CO_2_ levels via specific mechanisms of the vagus reflexes, molecular CO_2_ and pH effects. The effects of hypercapnia on the airways and airway smooth muscle is complicated. There are reports attesting to it causing increased airway contractility [26,76,77,78,79,80,81,82,83,84,85] or airway relaxation [86,87,88,89,90,91,92,93,94,95,96,97]. We review recent advances in our understanding of how elevated CO_2_ conditions modulate the airway tone, focusing on the effects of hypercapnia and respiratory acidosis.

#### 4.1.1. Hypercapnia

**Figure 1:**
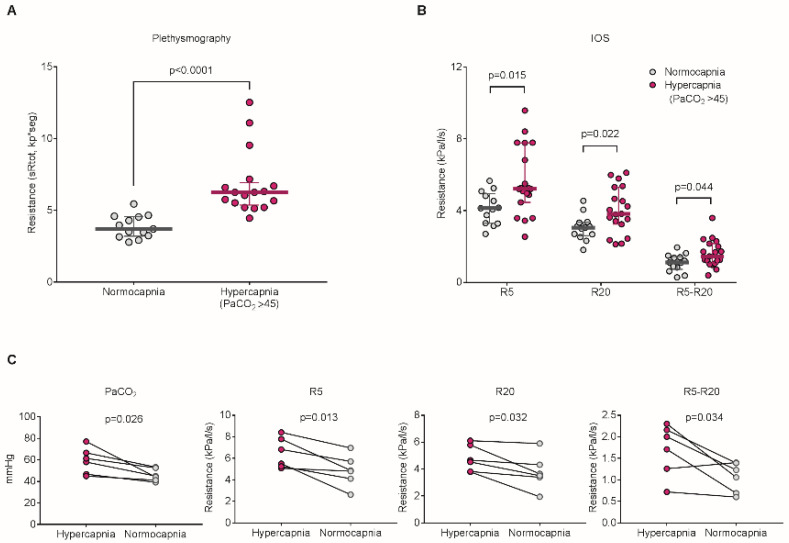
Hypercapnia increases airway and respiratory resistance in patients with chronic obstructive pulmonary disease (COPD). (**A**) Comparison of airway resistance (sR_tot_) measured by plethysmographic assessment between normocapnic and hypercapnic patients. (**B**) Comparison of respiratory resistance measured by impulse oscillometry (IOS) between normocapnic and hypercapnic patients. Values of R5, R20, and R5-R20 indicate total, proximal, and peripheral respiratory resistance, respectively. (**C**) Changes of respiratory resistance in hypercapnic patients. Reproduced from [26]. Copyright © 2018 American Association for the Advancement of Science.

Airway tone is regulated by interaction of the sympathetic and parasympathetic pathways [82,98] where the stimulation of vagal efferent nerves can increase bronchoconstriction [82,98,99,100]. Evidence suggesting that changes in CO_2_ levels in the blood affect the airway tone was first reported in 1892 [76]. Einthoven described that inhalation of high concentrations of carbonic acid (CO_2_-rich mixtures) caused bronchoconstriction in dogs, which was confirmed in various models of normoxic hypercapnia-exposed dogs [77,78,79,80,81] and cats [82,83]. The hypercapnia-induced bronchoconstriction was abolished by blocking the vagus nerve and understood to be dependent on the integrity of vagal conduction [76,77,78,79,80,82,83]. In healthy human volunteers, it has been reported that inhalation of high CO_2_ concentrations decreases specific airway or pulmonary conductance [84,85]. The increases in airway resistance during high CO_2_ exposure were initially interpreted as extrathoracic airway narrowing [84] such as larynx narrowing [85], because the hypercapnic effect was not blocked by atropine or β_1_/β_2_ adrenergic receptor agonists. However, the direct studies of laryngeal resistance during high CO_2_ exposure indicated no change in animal models [101] and normal human subjects [102]. Furthermore, several reports of bronchoconstriction in the hypercapnia-exposed animal models [78,80,82] revealed that the blockage of the vagus nerve did not entirely abolish the bronchoconstrictor response to the high CO_2_ exposure, suggesting that other mechanisms contribute to the hypercapnia-mediated airway constriction. More recently, we have reported that high CO_2_ acts as a signaling molecule to increase smooth muscle contraction in mouse and human airway smooth muscle cells [26]. We found that high concentrations of CO_2_, independently of hypoxia and extracellular pH, increased acetylcholine-induced cell contraction dependent on CO_2_ dose and exposure time in cell culture systems. In a murine model, the exposure to normoxic hypercapnia increased acetylcholine-induced airway contraction in precision lung cut slices as well as airway resistance. Furthermore, we found that, in a small cohort of patients with chronic obstructive pulmonary disease (COPD), patients with hypercapnia (PaCO_2_ > 45 mmHg) had higher airway resistance, which improved after correction of hypercapnia (Figure 1).

Our study also provided insights into the molecular mechanisms by which high CO_2_ levels promote airway smooth muscle cell contractility via calcium-calpain signaling. The signaling was mediated by caspase-7, which by cleaving the transcription factor myocyte-specific enhancer factor 2D (MEF2D), leads to downregulation of the microRNA-133a (miR-133a) and consequent upregulation of Ras homolog family member (Rho) A and myosin light-chain (MLC) phosphorylation. Our data suggest that elevated CO_2_ levels activate specific signal transduction pathways in airway smooth muscle cells, which results in deleterious effects on the airway tone, leading to bronchoconstriction. Taken together, these more recent reports suggest that hypercapnia promotes airway constriction by activating the vagus nerve and high CO_2_-responsive signal transduction pathways to worsen airway obstruction in patients with severe asthma.

#### 4.1.2. Respiratory Acidosis

Hypercapnia has been also reported to lead to airway relaxation [86,87,88,89,90,91,92,93,94,95,96,97]. Inhalation of high CO_2_ concentrations initially decreased airway constriction as well as the isolated bronchial ring tension caused by drugs such as 5-hydroxytryptamine [88,89,90]. It also reversed the airway constriction associated with pulmonary artery occlusion in ventilated animal models [86,88]. In humans, the administration of high CO_2_ relaxed the constriction of airways in a patient with unilateral pulmonary artery occlusion [87] and young asthmatic adults with hyperventilation [91] or exercise-induced bronchoconstriction [91,92]. These effects of hypercapnia were not mediated by the nerve reflexes and are understood to be due to changes in extracellular/intracellular pH levels, possibly acute respiratory acidosis in airway smooth muscle cells. Several in vitro studies reported that respiratory or normocapnic (metabolic) acidosis caused a reversible reduction in active tension of bronchial rings [89,90,93,94]. Extracellular pH can alter airway smooth muscle tone by changing the levels of intracellular pH and calcium (Ca^2+^) [94,95,103]. Intracellular acidification has been reported to decrease intracellular Ca^2+^ levels through voltage-dependent Ca^2+^ channels in the K^+^-induced contractile model, thereby inhibiting airway smooth muscle cell contraction [96]. We have reported that airway smooth muscle relaxation occurred during acute hypercapnia, but it was early, modest and transient [26]. As such, we reason that elevated CO_2_ levels may have a transient relaxing effect on contracted airways due to the decrease in pH, i.e., respiratory acidosis. There are no reports describing that hypercapnia and acute respiratory acidosis improved airway contractility or obstruction during acute exacerbation in patients with severe asthma.

### 4.2. Innate Immunity

Respiratory infection is one of the risk factors for development and exacerbation in patients with asthma [104,105,106,107]. Recent studies have reported that viral and/or bacterial infections were observed in 70% of adult inpatients with an asthma exacerbation [106]. Viral and bacterial super-infection is an important determinant of severe acute exacerbations and was more likely to result in hospital readmission following severe acute exacerbation [104]. Hypercapnia has been reported to be associated with increased mortality in hospitalized patients with community-acquired pneumonia [108] and in patients with cystic fibrosis awaiting lung transplantation [109]. In transcriptomic analyses of hypercapnia in model organisms, exposure to normoxic hypercapnia altered the expression of innate immune system genes in *Caenorhabditis elegans* [110] and *Drosophila melanogaster* [111]. In adult flies and the *Drosophila* S2 cell line, hypercapnia suppressed induction of genes involved in specific antimicrobial peptides such as diptericin that are regulated by Relish, an orthologue of the mammalian transcription factor NF-κB [111]. Transcriptomic studies in mouse neonatal lung tissue and human bronchial epithelial cells reported that hypercapnia altered the expression of components of the innate immune system [38,39]. It downregulated the expression of inflammatory mediator genes including interferons, interleukins, chemokines and tumor necrosis factor (TNF) in the neonatal lung [38]. In human bronchial epithelial cells, hypercapnic respiratory acidosis resulted in downregulation of genes related to the interleukin 6 (IL-6) receptor and chemokines [39]. Hypercapnia selectively inhibited the expression of IL-6 and TNF, and decreased phagocytosis in human and mouse alveolar macrophages [33]. Fitzpatrick et al., reported that in patients with moderate and severe asthma alveolar macrophage phagocytosis was decreased by more than 50% compared with that seen in control subjects and the impairment of phagocytosis was associated with poorly controlled asthma [112]. Hypercapnia also inhibited Beclin 1 activity by increases in Bcl-2 and Bcl-xL expression, and prevented autophagy and bacterial killing in human macrophages [36]. Furthermore, hypercapnia led to inhibition of the canonical NF-κB pathway while promoting activation of the noncanonical NF-κB component IKKα/RelB/p100, whose function is largely anti-inflammatory and immunosuppressive [34,35,113]. In these in vitro studies, the high CO_2_-induced inhibitions of cytokine gene expression, phagocytosis, autophagy and NF-κB signaling was independent of pH effects. Contrastingly, acidosis is known to impair the function of immune cells [114], including alveolar macrophages [115]. Thus, hypercapnia might modulate innate immunity and host defense via pH-independent or -dependent mechanisms. In mice exposed to normoxic hypercapnia, high CO_2_ levels decreased IL-6 and TNF expression in the lung during the early phase of *Pseudomonas aeruginosa* infection, and inhibited the phagocytosis of bacteria and generation of reactive oxygen species by lung neutrophils, resulting in increased burden of *Pseudomonas aeruginosa* in the lungs and other organs and higher mortality [25]. More recently, Casalino-Matsuda et al., has reported that hypercapnia increased virus-induced lung injury and mortality in mice infected with influenza A virus and hypercapnia [29]. They observed that elevated CO_2_ levels increased influenza A virus replication and inhibited antiviral gene and protein expression in macrophages. Interestingly, both in vivo studies showed similar reversibility of hypercapnia-induced defects in antiviral and antibacterial immunity [25,29]. There have been several reports suggesting that hypercapnia activates the renin-angiotensin system and angiotensin-converting enzyme 2 (ACE2) expression [116,117,118], which is identified as a receptor for the spike protein of SARS-CoV-2 and facilitating the viral entry into target cells [118]. It has not yet been reported that hypercapnia affects the pathogenesis of SARS-CoV-2 infection, but these findings in the studies of hypercapnia may also provide new insights into the understanding of the SARS-CoV-2 infection mechanisms. Elevated CO_2_ level should be taken into consideration as a potential risk factor to cause and worsen any infections in patients with severe asthma.

### 4.3. Adipogenesis

Asthma and obstructive sleep apnea (OSA) have been reported to coexist and contribute to an overlap syndrome where a bidirectional relationship may negatively affect the other condition [119,120,121]. A recent meta-analysis has revealed that the prevalence of OSA and OSA risk in adult asthmatic patients is 50% and 27.5%, respectively, and the odds of having OSA or OSA risk is 2.64 and 3.73 times higher in asthmatic patients than in non-asthmatic patients [122]. Asthmatic patients with OSA had significantly higher body mass index (BMI) in comparison with non-asthmatic patients [122]. Redline et al., reported that a 1 kg/m^2^ increase in BMI above the mean for age and sex translated to a 12% increase in risk of OSA [123], suggesting that OSA is related to obesity which is known to be prevalent in patients with severe asthma [124,125,126]. Obese patients with asthma have more severe disease with increased medication use [126] and a 4.6-fold higher risk of hospitalization as compared to the non-obese patients with asthma [127]. Obesity exacerbates OSA via several mechanisms; (1) neck adiposity decreases the size of the upper airway lumen, (2) abdominal adiposity decreases lung volumes and chest wall compliance, and increases airway resistance, (3) obesity-associated leptin resistance decreases ventilatory drive and response to hypercapnia [128,129]. Recently, Kikuchi et al., reported that either intermittent or sustained exposure to hypercapnia, independently of acidosis and oxygenation levels, promoted adipogenesis in visceral and subcutaneous preadipocytes [130]. The mechanisms by which hypercapnia induced adipogenesis lead to increased production of cyclic adenosine monophosphate (cAMP) via soluble adenylyl cyclase and activation of protein kinase A and exchanger protein directly activated by cAMP (EPAC). This, in turn, activates proadipogenic transcription factors, such as cAMP response element binding protein (CREB), CCAAT/enhancer binding protein β (C/EBP-β), and peroxisome proliferator–activated receptor γ (PPAR-γ). In addition, plasma leptin levels contribute to an aberrant hypercapnic ventilatory response in obese patients. Leptin is produced by adipocytes and its levels in serum correlate positively with total body fat mass [131]. In animal models, it has been reported that leptin-deficient mice show a blunted ventilator response to hypercapnia, suggesting that leptin can act as the respiratory stimulus [132,133]. In contrast, obese patients show a relative deficiency of leptin in the cerebrospinal fluid as compared with lean controls [134], suggesting a failure of central feedback mechanisms, “leptin resistance”. These data are supported by evidence linking hyperleptinemia and reduced respiratory drive and hypercapnic response to leptin resistance of the respiratory center [135]. Collectively, these reports suggest a maladaptive cycle between hypoventilation, hypercapnia and increased fat mass, leading to the progression of obesity and OSA, thus contributing to asthma severity.

## 5. Conclusions

**Figure 2:**
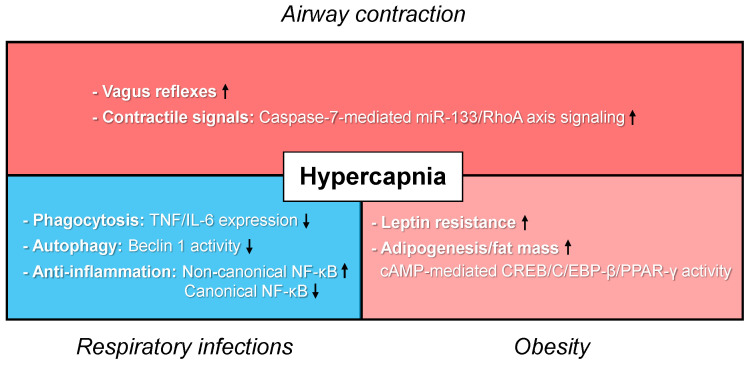
Schematic summarizing potential detrimental effects of hypercapnia in patients with severe asthma. Recent discoveries indicate that hypercapnia increases airway contractility, impairs the innate immune response, and promotes adipogenesis, which likely underlies the negative effects of elevated carbon dioxide (CO_2_) on airway contraction, respiratory infections and obesity in patients with asthma.

Hypercapnic respiratory failure is a hallmark of severe asthma. As reviewed above, elevated CO_2_ levels are rapidly sensed by chemosensing regions of the brain that regulate the respiratory drive, however, there are many reports suggesting that hypercapnia is also sensed by non-excitable cells and has significant effects on cellular and tissue functions. Recent discoveries suggest that hypercapnia increases airway contractility, impairs the innate immune response, and promotes adipogenesis, which likely underlies, at least in part, the negative effects of elevated CO_2_ in patients with asthma (Figure 2). Mechanical ventilation with “permissive hypercapnia” for severe asthma is currently an accepted therapeutic strategy [22,70]. The “permissive hypercapnia” approach is based on observational reports from the 1980s to 1990s [9,10,18,21] and is generally well tolerated for short periods of time if oxygenation is preserved and severe respiratory acidosis is avoided [70]. However, more recent studies challenge the “permissive hypercapnia” approach in view of new evidence suggesting that hypercapnia is harmful [23,26,27,136,137]. In recent years, noninvasive positive pressure ventilation (NPPV) aimed at correcting elevated PaCO_2_ values has been shown to be beneficial in patients with obstructive lung diseases and hypercapnia [26,136,137,138,139]. The institution of NPPV in hypercapnic patients with COPD improved outcomes: mortality, pulmonary function and health related quality of life [26,136,137]. In patients with asthma, a recent analysis of a national database documented increasing use of NPPV for life-threatening asthma and a concomitant decrease in use of invasive mechanical ventilation [140]. There is growing evidence reporting beneficial effects of NPPV by reducing hypercapnia, which is associated with improved pulmonary function and reduction in in-hospital mortality in asthmatic patients [138,139]. Recent preclinical and clinical studies of hypercapnia describe the mechanisms that underlie the benefits of reducing hypercapnia. Our review has summarized the data that could provide a guidance for re-assessment of the current paradigms of treatment and management in patients with asthma and hypercapnia.
